# Production of Japanese Encephalitis Virus Antigens in Plants Using Bamboo Mosaic Virus-Based Vector

**DOI:** 10.3389/fmicb.2017.00788

**Published:** 2017-05-03

**Authors:** Tsung-Hsien Chen, Chung-Chi Hu, Jia-Teh Liao, Yi-Ling Lee, Ying-Wen Huang, Na-Sheng Lin, Yi-Ling Lin, Yau-Heiu Hsu

**Affiliations:** ^1^Graduate Institute of Biotechnology, National Chung Hsing UniversityTaichung, Taiwan; ^2^Institute of Biomedical Sciences, Academia SinicaTaipei, Taiwan; ^3^Institute of Plant and Microbial Biology, Academia SinicaTaipei, Taiwan

**Keywords:** bamboo mosaic virus-based vector, chimeric virus particles (CVPs), foot-and-mouth disease virus 2A, Japanese encephalitis virus, vaccine, plant-made

## Abstract

Japanese encephalitis virus (JEV) is among the major threats to public health in Asia. For disease control and prevention, the efficient production of safe and effective vaccines against JEV is in urgent need. In this study, we produced a plant-made JEV vaccine candidate using a chimeric virus particle (CVP) strategy based on bamboo mosaic virus (BaMV) for epitope presentation. The chimeric virus, designated BJ2A, was constructed by fusing JEV envelope protein domain III (EDIII) at the N-terminus of BaMV coat protein, with an insertion of the foot-and-mouth disease virus 2A peptide to facilitate the production of both unfused and epitope-presenting for efficient assembly of the CVP vaccine candidate. The strategy allowed stable maintenance of the fusion construct over long-term serial passages in plants. Immuno-electron microscopy examination and immunization assays revealed that BJ2A is able to present the EDIII epitope on the surface of the CVPs, which stimulated effective neutralizing antibodies against JEV infection in mice. This study demonstrates the efficient production of an effective CVP vaccine candidate against JEV in plants by the BaMV-based epitope presentation system.

## Introduction

Japanese encephalitis virus (JEV), the causal agent of Japanese encephalitis (JE), is a plus-strand RNA virus of the family *Flaviviridae* (Vaughn and Hoke, 1992; Unni et al., 2011). JE is a major public health problem in Asia, causes up to 50,000 encephalitis cases and 10,000 deaths annually in humans (Campbell et al., 2011; Unni et al., 2011; Li et al., 2014; Tarantola et al., 2014; Cappelle et al., 2016). With the lack of specific antiviral treatment, vaccination against JEV is crucial for prevention (Li et al., 2014), and is recommended by the World Health Organization (WHO) for the at-risk populations (WHO, 2015). However, the successful implementation of vaccination programs in such areas may depend largely on the cost-effectiveness and safety concerns of the vaccines, similar to the cases for a close relative of JEV, the West Nile virus (Zohrabian et al., 2006; Martina et al., 2010; Chen, 2015).

Currently inactivated JEV vaccines prepared from infected mouse brains (BIKEN or JEVAX) or primary hamster kidney cells and a live attenuated vaccine (SA14-14-2) have been successfully developed to control JEV infection (Mackenzie et al., 2004; Ghosh and Basu, 2009). Nevertheless, the use of inactivated JEV vaccine does not confer sufficient long-term immunity to provide effective protection (Mackenzie et al., 2004; Ghosh and Basu, 2009). In addition, there are also concerns of side effects (Shlim and Solomon, 2002). Accordingly, WHO has designated JEV vaccines as a high-priority target for development of a new vaccine to fight against JE worldwide (Tsai, 2000).

The applications of plants as bioreactors to produce valuable proteins, including vaccines, have attracted considerable interests in recent years (Takeyama et al., 2015). Plants can produce large volumes of products efficiently and can have significant advantages in decreasing manufacturing costs (Thomas et al., 2011; Moustafa et al., 2016). The production of foreign proteins can be achieved through stable transformation of the nuclear or chloroplast genomes, or the transient expression mediated by *Agrobacterium*- or virus-based vector systems (Lico et al., 2008; Chen and Lai, 2013). Among these commonly used approaches, virus-based transient expression vector systems are particularly promising for rapid expression of recombinant proteins at levels higher than with stable transgenic plants (Daniell et al., 2009).

Plant viral vector systems explore various strategies for recombinant protein expression, including gene insertion or substitution, modular or deconstructed vector design, and protein fusion (peptide display) (Lico et al., 2008). The presentation of heterologous epitopes on plant virus particles is very convenient for peptide-based production of therapeutics and vaccines. The protein fusion strategy has been used extensively to display target peptides on the surface of chimeric virus particles (CVPs) to enhance immunogenicity (e.g., Gerloni et al., 2000; Smith et al., 2006; Massa et al., 2008; Hassani-Mehraban et al., 2015), and to facilitate easy antigen purification. In a previous study, we have reported the use of a bamboo mosaic virus (BaMV)-based vector as an effective epitope presentation system, and demonstrated that the foot-and-mouth disease virus (FMDV) VP1 epitopes expressed on BaMV CVPs can effectively induce humoral and cell-mediated immune responses in swine and provide full protection against FMDV challenges in that host (Yang et al., 2007). This BaMV-based CVP vector system presents an alternative approach for the development of a vaccine candidate against JEV.

Japanese encephalitis virus RNA contains a single open reading frame (ORF) that codes for a polyprotein which is proteolytically processed into three structural proteins designated envelope (E), membrane (M), and capsid (C) and seven non-structural proteins, NS1, NS2A, NS2B, NS3, NS4A, NS4B, and NS5 (Unni et al., 2011). The E protein appears to play an important role in viral attachment, membrane fusion for entry into the host cell (Stiasny and Heinz, 2006), virus assembly and maturation, and most notably, inducing virus-neutralizing antibodies (Mason et al., 1989; Kurane, 2002). The key domain of E protein, EDIII, forms a β-barrel type structure resembling the immunoglobulin constant domain and can be independently folded as an individual fragment by forming a disulfide bond (between residues 304 and 335) to maintain its conformation (Wu K. P. et al., 2003). Moreover, neutralizing epitopes in the EDIII have been identified on the lateral surface (Cecilia and Gould, 1991; Seif et al., 1995; Lin and Wu, 2003). Therefore, EDIII represents a potential antigen for producing vaccine candidates.

The use of autonomously replicating viruses as expression vectors provides an attractive means for the transient expression of CVPs displaying JEV EDIII antigens in plants. However, the sizes of the epitope presented in our previous BaMV-based CVP vector was limited to 37 amino acids (Yang et al., 2007), which is also a common barrier encountered by other CVP-based expression systems (e.g., Bendahmane et al., 1999; Jiang et al., 2006; Uhde-Holzem et al., 2010; Zhang et al., 2010). For epitopes with larger sizes, such as the EDIII epitope of JEV, or other unfavorable structural features, alternative strategies are required to improve the survival rate and the stability of the fusion proteins.

In this study, we aimed to develop a BaMV-based CVP vaccine against JEV by fusing JEV EDIII to BaMV coat protein (CP) and displaying the EDIII epitopes on the surfaces of CVPs. To overcome the size-limitations of the epitope-presentation systems, we have adopted the strategy of Cruz et al. (1996) by inserting the 2A co-translational dissociation sequence from FMDV (designated 2A) to the junction of JEV EDIII and BaMV CP, providing enhanced solidity of the CVPs while retaining the presentation of EDIII epitopes on portions of virion surfaces. Detailed analysis were performed to investigate the genetic stabilities of the chimeric virus and the proportions of EDIII-2A-BaMV CP fusion proteins assembled into CVPs among those produced in plant cells. Immunization assays were also conducted to examine the effectiveness of these chimeric CVPs to stimulate the immune responses in mice. Evidence was provided to support that the BaMV-based CVP may offer an alternative vaccine candidate to elicit the generation of neutralization antibodies in mice.

## Materials and Methods

### Construction of Chimeric BaMV Infectious Clone

**FIGURE 1:**
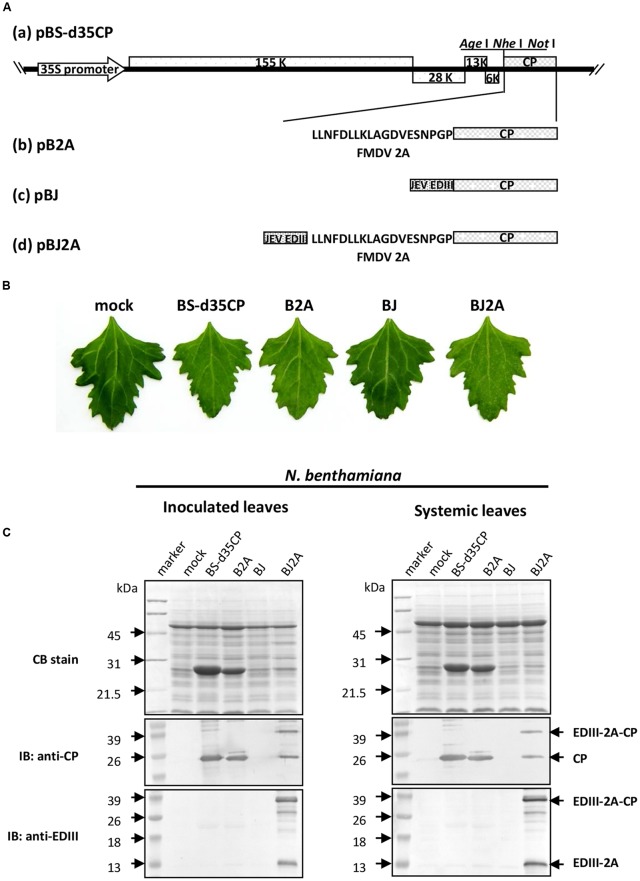
**Japanese encephalitis virus (JEV) EDIII is expressed in plants infected with chimeric BaMV. (A)** Schematic representation of the recombinant constructs based on BaMV genome. **(B)** Infectivity and symptom of various recombinant BaMV construct on *Chenopodium quinoa*. Leaves inoculated with H_2_O (mock) or recombinant plasmids pBS-d35CP, pB2A, pBJ, or pBJ2A were shown. The photos were taken at 10 days post-inoculation (dpi). **(C)** SDS-PAGE separation and immunoblot analysis of proteins extracted from inoculated or systemically infected leaves of *N. benthamiana*, as indicated on top of each panel. Leaves were H_2_O-inoculated (mock) or inoculated with recombinant plasmids pBS-d35CP, pB2A, pBJ, or pBJ2A as indicated. Total proteins extracted from inoculated leaves (accounting for 1 mg fresh weight of leaf) from each treatment were separated in a 12% SDS-PAGE (Top panel), and stained with coomassie blue (CB). The proteins were transferred to PVDF membranes and reacted with antisera against BaMV CP (anti-CP, middle panel), or JEV EDIII (anti-EDIII, bottom panel), respectively. The relative molecular weights (in kDa) are given on the left of each panel, and positions of each target proteins on the right. IB, immuno-blot.

The infectious recombinant constructs used in this study were derived from a mutant BaMV cDNA plasmid, pBS-d35CP (Yang et al., 2007) (**Figure 1A**), in which the N-terminal 35 amino acids of CP have been deleted. The coding sequence of JEV (CH2195LA strain) EDIII region, from nucleotide position 874 to 1206, was amplified with primers 5′ ggactagtaccatg*gacaaactggccctgaaaggc* 3′ and 5′ cgttccagctccagacattgcggccgc*cgtgcttcctgctttgtg* 3′ (with JEV EDIII coding sequences italicized, and restriction sites for *Spe*I, and *Not*I, respectively, underlined) by PCR using plasmid pET32a/LD3 (Wu S. C. et al., 2003) as the template. The PCR-amplified fragment was purified and inserted into plasmid pBS-d35CP at the *Nhe*I and *Not*I site, resulting in plasmid pBJ (**Figure 1A**). The DNA fragment coding for the FMDV 2A peptide (LLNFDLLKLAGDVESNPGP) (Ryan et al., 1991) was amplified by PCR with primers 5′ ggctagcgcggccgcg***c****tgttgaattttga****ccttcttaagcttgcggg*** 3′ and 5′ cctgggcccc*gggtccggggttggactcgacgtct****cccgcaagcttaagaagg*** 3′ (with FMDV 2A coding sequence italicized, restriction sites underlined for *Nhe*I, *Not*I, in conjunction, and *Psp*OMI, respectively, and complementary sequences in boldface). Plasmid pB2A was constructed by inserting the FMDV 2A coding sequence at the 5′-terminus of the truncated CP ORF of pBS-d35CP with proper restriction enzyme digestions (**Figure 1A**). The above-mentioned JEV EDIII coding sequence was inserted into plasmid pB2A at the *Nhe*I and *Not*I site to give plasmid pBJ2A (**Figure 1A**). The identities of all plasmids were confirmed by nucleotide sequencing.

### Preparation of Recombinant EDIII (rEDIII)

Japanese encephalitis virus EDIII fragments were obtained from pET32a/LD3 plasmid (Wu S. C. et al., 2003) by digestion with *Nco*I and *Not*I, and cloned into plasmid pET21d (Novagen) at the respective sites for over-expression in *Escherichia coli*. Methods used for expression and purification of rEDIII protein were as previously reported (Seif et al., 1995), except that the *E. coli* strain BL21(DE3) (Novagen) was transformed with the rEDIII-expression plasmid and grown overnight in LB medium in the presence of ampicillin (50 μg ml^-1^). The cells were then diluted 50-fold in LB medium containing ampicillin and grown at 37°C. The rEDIII protein was further dialyzed against phosphate-buffered saline (PBS). The purified rEDIII was further subjected to raise specific antiserum in rabbits following standard procedures (Lin and Chen, 1991).

### Protein Analysis of the Infected Plant Tissue and Stability of Chimeras during Sequential Transmission

The genetic stability of BJ2A chimeric virus was tested using local-lesion host *Chenopodium quinoa*, while the systemic movement of the chimeric virus was tested on systemic-infection host *Nicotiana benthamiana*. The infectious viral cDNA clones of pBS-d35CP, pB2A, pBJ, and pBJ2A were inoculated onto *N. benthamiana* or *C. quinoa* as previously reported (Yang et al., 2007). The plants were grown in a greenhouse exposed to normal daylight. After local lesions appeared on the pBJ2A-inoculated leaves of *C. quinoa* at 10 days post-inoculation (dpi), leaves were excised and ground in deionized H_2_O (1:10; weight:volume). The crude sap was mechanically inoculated to healthy *C. quinoa*. The above-mentioned procedure was repeated for nine times, and the progeny virus, BJ2A, on *C. quinoa* leaves was assayed each time to examine the stability of the chimeric virus during successive passages in plants. Total proteins extracted from inoculated leaves were separated by electrophoresis on a 12% polyacrylamide gel containing 1% sodium dodecyl sulfate (SDS-PAGE), and stained with coomassie blue (CB). The proteins were then transferred to PVDF membranes (Millipore) and reacted with antisera against BaMV CP (Lin and Chen, 1991) or rEDIII, respectively.

### Detection of EDIII in Inoculated Plants by Enzyme-Linked Immunosorbent Assay (ELISA)

The plant-made JEV EDIII proteins in *C. quinoa* leaves inoculated with pBJ2A were examined by indirect ELISA using the rabbit antiserum against rEDIII. ELISA was performed as described previously with minor modifications (Saejung et al., 2007). The bound protein-antibodies were detected with biotin-conjugated goat anti- rabbit IgG using the VECTASTAIN Elite ABC kit (avidin biotinylated peroxidase; Vector Laboratories). Following color development, the absorbance at 450 nm was measured on an ELISA reader (Spectramax M2, Molecular Device, USA). Known amounts of purified JEV rEDIII protein was used to establish the standard curve for quantification. Protein extract from a healthy plant was used as a negative control.

### BJ2A CVP Purification

*Chenopodium quinoa* was chosen as the host plant for the production of BJ2A CVPs to avoid the potential side effects of nicotine and other alkaloids present in *N. benthamiana* (Mishra et al., 2015). Leaves of *C. quinoa* inoculated with pBJ2A were harvested at 10 dpi. The BJ2A CVPs were subsequently purified from the leaves and the yield was determined spectrophotometrically by absorbance at 280 nm as described previously (Lin and Chen, 1991). Purified BJ2A CVPs were dissolved in BE buffer (50 mM Borate, pH 8.0, 1 mM EDTA), then stored at -20°C until used. Chimeric BJ2A virions were separated on a 12% SDS-PAGE. The protein bands corresponding to the EDIII-2A-CP fusion protein or cleaved CP on the gel were quantitated by using the Alpha Imager 2200 V5.04 documentation and analysis system.

### Immunoelectron Microscopy

Methods used for the examination of chimeric BJ2A virions by immunoelectron microscopy were as previously reported (Lin, 1984). Gold-labeled antibodies specific for BaMV CP (Lin and Chen, 1991), JEV EDIII, and pre-immune serum were used in the respective experiments. The grids were finally negatively stained with 2% uranyl acetate and examined with transmission electron microscopy (Philips CM 100 Bio) at 80 KV. Control grids were treated with pre-immune rabbit antiserum.

### Mouse Immune Response

Three groups of 6-week-old female BALB/c ByJ mice, six mice per group, obtained from the National Laboratory Animal Center (Taipei, Taiwan), were immunized by intraperitoneal injection. The care of the animals was provided in accordance with guidelines approved by the animal committee of the Institute of Biomedical Sciences, Academia Sinica. One group was immunized with 200 μg BJ2A CVPs. The second group was immunized with 30 μg rEDIII as the positive control. The third group was injected with saline as the negative control. All the mice were boosted with the same dose on day 12. The primary antigens were emulsified in Freund’s complete adjuvant (Difco) and boosters emulsified in Freund’s incomplete adjuvant (Sigma). Sera were collected on days 0 and 49. The titers and reactivity of sera were tested using indirect ELISA, indirect immunofluorescence assay and plaque reduction neutralization, described as follows.

### Analysis of EDIII-Specific Antibody in Mice Sera by ELISA

Serum samples were collected by periorbital route and heat-inactivated at 56°C for 30 min. JEV EDIII-specific antibodies in serum samples were analyzed by indirect ELISA as described previously (Yang et al., 2007), except that ELISA plates (Nunc) were coated with rEDIII (1 μg per well) as antigens, and bound antibodies were detected with biotin-conjugated goat anti-mouse IgG (H+L). Following color development, the absorbance at 450 nm was measured on an ELISA reader. For background reactions, mice pre-immune sera were used in the ELISA.

### Indirect Immunofluorescence Assay

To analyze whether the BJ2A CVPs elicited the production of effective JEV EDIII-specific antibodies in the immunized mice, indirect immunofluorescence assay was performed as described previously (Wu et al., 2002), except that BHK-21 cells were infected with JEV (RP-9 strain) and the sera obtained from the immunized mice were pooled and 100-fold diluted. Fluorescence was observed with a Leica fluorescence microscope. Cell nuclei were visualized by 4, 6-diamidino-2 -phenylindole (DAPI) staining in 0.9% sodium chloride. Pictures were taken using an inverted fluorescent microscope (Leica) by double exposure of the same fields with filters for FITC and DAPI.

### Neutralization Test

Neutralizing antibody was assayed by plaque reduction neutralization test (PRNT) in BHK-21 cells as previously described (Chen et al., 2005) with minor modifications. Briefly, serum samples were subjected to a serial twofold dilution in 5% fetal bovine serum (FBS)-PBS on ice. Then, equal volumes of infectious JEV in minimum essential medium (MEM) supplemented with FBS were mixed with the serially diluted serum sample to make a mixture containing approximately 100 pfu of virus per well. The virus-antibody complex was added to six-well plates (in triplicates) containing confluent monolayers of BHK-21 cells. The plates were incubated at 37°C for 1 hr with gentle rocking every 15 min. The wells were then overlaid with 2 ml of 1% methyl cellulose prepared in MEM, supplemented with 5% FBS and incubated at 37°C in 5% CO_2_ for 4 days. Plaques were stained with naphthol blue black and counted. The neutralizing antibody titer was calculated as the reciprocal of the highest dilution resulting in a 70% reduction of plaques compared to that of a control of virus without antibody added.

## Results

### Production of JEV EDIII Using Chimeric BaMV Vectors in Plants

To achieve better yield and stability of JEV EDIII in plants, we explored two different strategies by using BaMV-based vector: (i) direct fusion of JEV EDIII to the N-terminus of truncated BaMV CP, and (ii) insertion of FMDV 2A co-translational dissociation peptide sequence in between JEV EDIII and BaMV CP. The first approach was expected to result in higher yield of the epitope, with JEV EDIII presented on every BaMV CP subunits, at the cost of losing virion stability. The second approach allowed for the production of both the JEV EDIII-2A-BaMV CP recombinant protein and the unfused BaMV CP, leading to the display of JEV EDIII on only portions of the chimeric BaMV virions, with the expected increase in stability of the CVP. Accordingly, two recombinant plasmids, pBJ and pBJ2A, were constructed based on a modified BaMV vector pBS-d35CP (**Figure 1A**). The infectivity of the recombinant viral vectors was assayed in both *N. benthamiana* and *C. quinoa*. The result revealed that infection with pBJ2A led to stronger mosaic symptoms than that with pBS-d35CP in *N. benthamiana*, whereas chlorotic local lesions distinct from those caused by pBS-d35CP were observed after pBJ2A inoculation in *C. quinoa* (**Figure 1B**). In contrast, inoculation with pBJ did not cause any visible symptom on both *N. benthamiana* and *C. quinoa* (**Figure 1B**).

To determine whether fusion proteins were produced properly in plants inoculated with the chimeric viruses, total proteins from the inoculated leaves of *N. benthamiana* infected with distilled water (mock), pBS-d35CP, pB2A, pBJ, or pBJ2A were subjected to analyses by SDS-PAGE (**Figure 1C**) and western blotting assay using BaMV CP-specific antibodies (**Figure 1C**, middle panel). As anticipated, no BaMV CP was detected in protein extract of mock-inoculated leaves (**Figure 1C**, mock). The FMDV 2A-BaMV CP fusion protein and N-terminal 35-amino-acid truncated BaMV CP (**Figure 1C**, middle panel, B2A) were both detected in the protein extract of pB2A-inoculated leaves, which migrated slightly faster than the chimeric CP from pBJ2A-inoculated leaves (**Figure 1C**, middle panel, BJ2A). In contrast, no BaMV CP was detected in the pBJ-inoculated leaves (**Figure 1C**, middle panel, BJ). To further verify that chimeric CP generated from pBJ2A-inoculated leaves harbored JEV EDIII peptide, western blotting analysis using rEDIII-specific antiserum was performed (**Figure 1C**, lower panel). Two proteins of 36.8 and 14.1 kDa were detected by rEDIII-specific antiserum, corresponding to the chimeric EDIII-2A-CP fusion protein and the free JEV EDIII, respectively (**Figure 1C**, lower panel, BJ2A). In contrast, no protein band was detected by the rEDIII-specific antiserum in protein extracts from leaves inoculated with pBS-d35CP, pB2A, or pBJ (**Figure 1C**, lower panel). Similar results were obtained when total protein extracts from systemic leaves of the infected *N. benthamiana* were assayed by western blotting using either BaMV CP- or rEDIII-specific antisera, respectively (**Figure 1C**, right panel). The above results suggested that the incorporation of FMDV 2A peptide did not affect the replication and systemic movement of the chimeric viruses, and indeed improved the infectivity of pBJ2A as compared to pBJ in both host plants tested.

### Stability of BJ2A Chimeras during Successive Passages in *C. quinoa*

**FIGURE 2:**
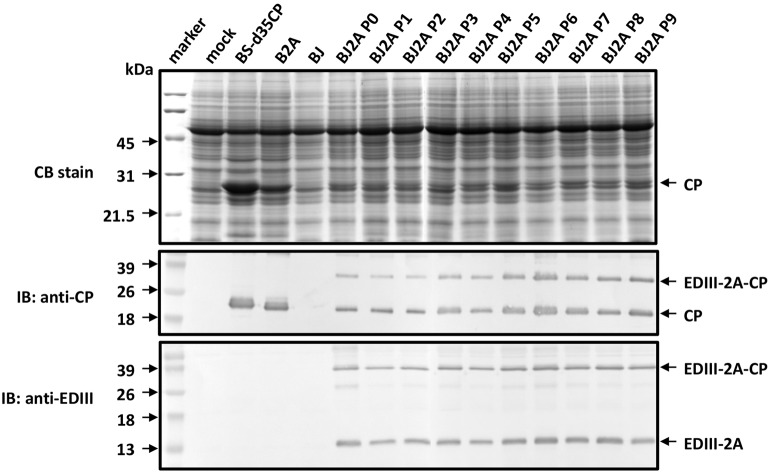
**Analysis of the stability of the chimeric BJ2A over serial passages in *C. quinoa* plants by SDS-PAGE and immunoblot.** Leaves were H_2_O-inoculated (mock) or inoculated with recombinant plasmids pBS-d35CP, pB2A, pBJ, or pBJ2A, respectively. BJ2A P0 denotes the initial inoculation with the plasmid DNA as inoculum, whereas P1to P9 indicate the 1st to 9th passage using crude leaf sap from P0 as inocula, respectively. SDS-PAGE and immunoblot assays were performed as described in **Figure 1C**.

To examine the stability of the chimeric BJ2A virus during successive passages in plants, infectious recombinant pBJ2A plasmid was inoculated onto *C. quinoa* leaves to generate the initial inoculum, designated P0, which was then subjected to nine sequential transmissions (P1 through P9) in *C. quinoa*. The presence of the EDIII-2A-CP fusion protein was monitored at each transfer by western blot analysis using specific antisera. All inoculated leaves developed lesions similar in appearance and number to those observed on P0-infected plants. Results from western blot analyses using antisera specific to BaMV CP or JEV EDIII clearly identified fusion-form BJ2A CP (36.8 kDa), EDIII2A polyprotein (14.1 kDa) and free CP (22.7 kDa) in total protein extracts from all the serially inoculated *C. quinoa* plants (**Figure 2**). After quantification of the proteins by using ELISA, the level of JEV EDIII expressed in the leaves of the *C. quinoa* plants was estimated to be 8.9 ± 4.3 μg mg^-1^, corresponding to 0.89 ± 0.43% of total soluble protein (TSP). The result demonstrated that the insertion of foreign coding sequences could be stably maintained in the genome of the chimeric virus over serial passages.

### JEV EDIII Peptides on the Outer Surfaces of CVPs

**FIGURE 3:**
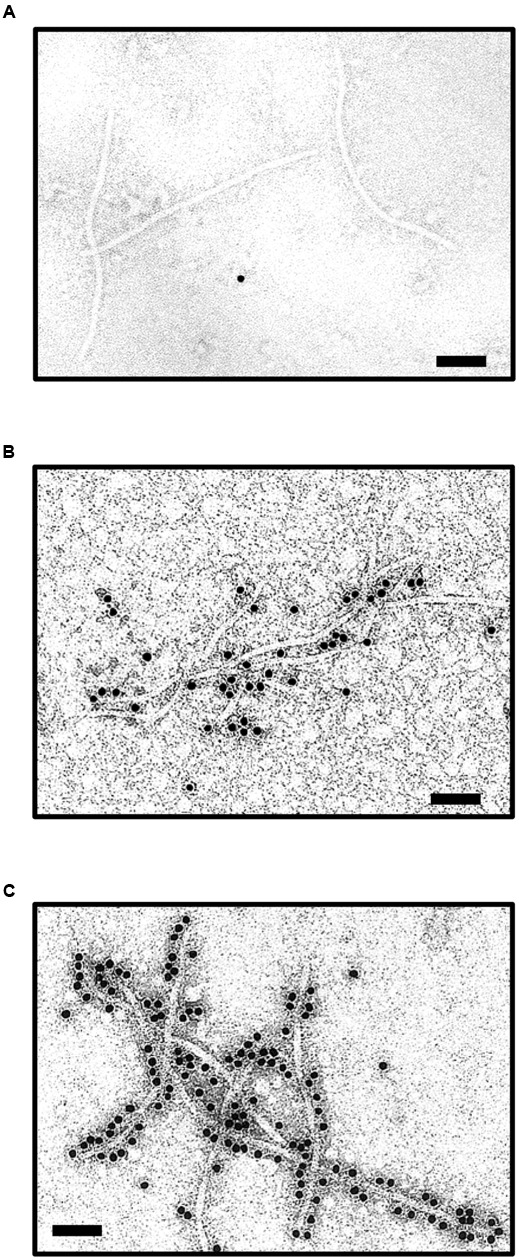
**Immunoelectron microscopy for the identification of JEV EDIII on the surface of BJ2A virus particles.** Purified BJ2A virions were incubated with pre-immune rabbit antiserum **(A)**, or antisera specific for JEV EDIII **(B)** or BaMV CP **(C)**, followed by gold-labeled goat anti-rabbit IgG secondary antibody, and subjected to examination by transmission electron microscopy. Scale bars, 100 nm.

Following the successful observation of the stable expression of both BJ2A fusion proteins and free CP in the inoculated plants, it is important to examine whether the CVPs could be properly assembled with the EDIII peptides presented on the outer surfaces. Results of the electron microscopy observation revealed that the BJ2A CVPs appeared typically filamentous with lengths approximately the same as those of the wild type BaMV virions (480 mm) (**Figures 3A–C**). Immunogold labeling using polyclonal antibodies against EDIII confirmed that the foreign EDIII epitopes were accessible and exposed on the surface of the CPVs (**Figure 3B**). As controls, BJ2A CVPs were labeled with gold-conjugated antiserum against BaMV CP (**Figure 3C**), but not with pre-immune serum (**Figure 3A**). The results demonstrated that the CPs and EDIII-2A-CP fusions can be properly assembled into BJ2A CVPs.

### Induction of Anti-JEV Antibody in Mice Immunized with Purified BJ2A CVPs

**FIGURE 4:**
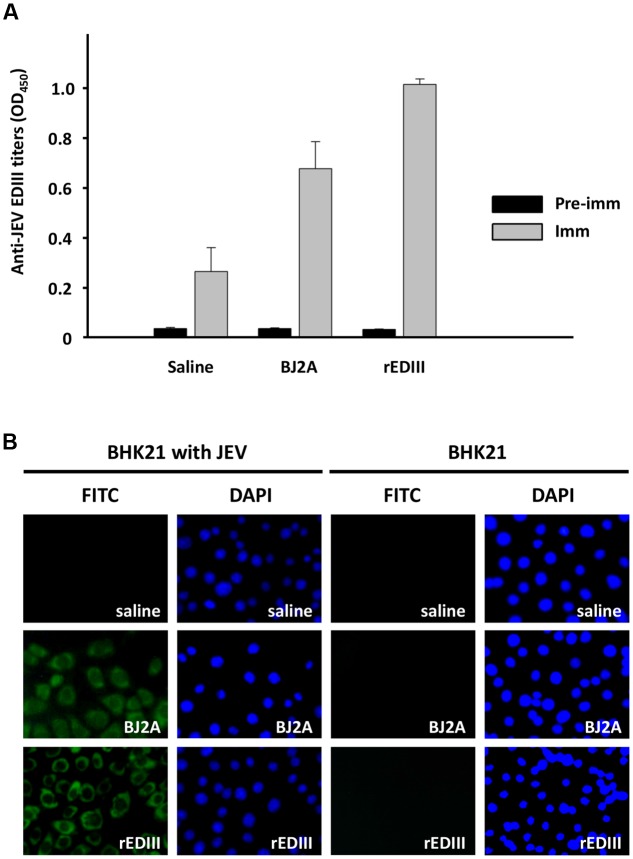
**Immune response of mice injected intraperitoneally with chimeric virus BJ2A as a vaccine candidate. (A)** Determination of immune response by ELISA. Sera from mice immunized with saline, BJ2A, or rEDIII, were subjected to ELISA using rDEIII as the antigen. The titers of antisera were determined by blocking ELISA as described (Yang et al., 2007). The columns represent the mean O.D. (at 450 nm) values obtained with sera from individual mice with standard deviations shown as error bars. **(B)** Analysis of effective JEV EDIII-specific antibodies by indirect immunofluorescence assay. BHK-21 cells, infected with JEV or uninfected as indicated on the top, were fixed and stained with pooled sera prepared from mice immunized with saline, BJ2A, or rEDIII and an FITC-conjugated secondary antibody, followed by examination with an inverted fluorescent microscope (Leica) (panels denoted by “FITC”). Cell nuclei were stained by DAPI (panels denoted by “DAPI”).

To determine immunogenicity and efficacy of the CVPs with target proteins presented on the surface, immune responses in mice were assayed as describes in section “Materials and Methods.” Blood samples from each group were collected from the periorbital route at days 0 and 49 after immunization. The reactivity to JEV EDIII by sera from BJ2A CVPs-immunized mice was examined by ELISA. The result showed that BJ2A-immunization elicited high levels of anti-EDIII antibodies in sera of the treated mice, similar to those observed for sera from rEDIII-immunized mice as a positive control (**Figure 4A**). The antibody reactivity was weak in the negative control group, which received a combination of saline and adjuvant throughout the experiment (**Figure 4A**). Subsequently, the reactivity of BJ2A CVP-immunized sera was tested by immunofluorescence assay in JEV infected BHK 21 cells. The results showed that sera from BJ2A CVP- or rEDIII-immunized mice recognized the JEV infected BHK 21 cells (**Figure 4B**), demonstrating their *ex vivo* reactivity. As a negative control, no fluorescence was detected when using sera from the group that received a combination of saline and adjuvant throughout the experiment, nor in non-infected BHK 21 cells (**Figure 4B**).

To further demonstrate the potential of the BJ2A CVPs as a vaccine candidate, JEV-specific neutralizing antibodies were measured by PRNT, which provides a reasonable immunogenic correlation to protection (Chen et al., 2005). Neutralization efficacy was determined by PRNT 70 titer (serum dilution giving a 70% plaque reduction compared with plaque formation in virus-only controls). Seroconversion was defined as a fourfold or greater increase in PRNT 70 titer (WHO, 2005). Indeed, a fourfold increase of JEV-specific neutralizing antibody titers were detected in pooled sera from mice immunized with BJ2A CVPs (PRNT 70 = 1:160) than from those immunized with saline (**Table 1**). The result suggested that the BJ2A CVPs could elicit effective immunity against JEV infections.

**Table 1:** Plaque reduction neutralization titers of sera obtained from mice immunized with BJ2A chimeric virus particles.

Immunogen	Plaque neutralization titers^∗^
Saline	1:40
BJ2A	1:160
rEDIII	1:320


^∗^*Represented as the serum dilution yielding 70% reduction in plaque number.Pooled sera from six mice were used for the study. Sera from mice immunized with saline or rEDIII were used as negative and positive controls, respectively.*

## Discussion

### Circumventing the Epitope Size-Limitation Problems for Virus-Based Vector Systems by the Incorporation of FMDV 2A Peptide

The use of plants as safer and less expensive production systems for vaccine antigens has been actively investigated for more than 20 years (Rybicki, 2010), including several plant virus-based expression systems (for excellent reviews, see Lico et al., 2008; Rybicki, 2014; Chen, 2015; Streatfield et al., 2015; Shahid and Daniell, 2016). The CP genes of viruses are commonly exploited for the development of various strategies, since CP genes are usually expressed with high efficiency and provide natural scaffoldings for the target proteins to be displayed on the surface of CVPs (Lico et al., 2008). However, the production of antigens using plant viral vectors is hindered by several common limitations that stem from the interference of the normal biological functions of viral proteins by the fused peptides. These problems include: (1) the reliability of epitope presentation affected by the nature and sizes of foreign peptides (e.g., Bendahmane et al., 1999; Jiang et al., 2006; Uhde-Holzem et al., 2010; Zhang et al., 2010); (2) mutual restriction between encoding recombination virus RNA and the chimeric CP (e.g., Rao, 2006; Schneemann, 2006), and virus-host interactions (e.g., Porta et al., 2003; Ahlquist et al., 2005; Chen et al., 2007); (3) the stability of the foreign fragments over long-term successive passages (e.g., Porta and Lomonossoff, 1998; Porta et al., 2003; Lico et al., 2006); (4) reduced efficiency for virion assembly caused by special structural features of the chimeric CP (e.g., Canizares et al., 2005), and (5) the changes in virion morphology and stability due to cysteine residues in the foreign peptide (e.g., Li et al., 2007). Likewise, the construct pBJ, harboring direct fusion between JEV EDIII and BaMV CP, was not infectious, and the fusion protein was not detected in inoculated plants (**Figures 1C, 2**). In this study, we presented several lines of evidence that these obstacles were circumvented by the incorporation of FMDV 2A co-translational dissociation peptide in between JEV EDIII and BaMV CP. The resulting construct, pBJ2A, was infectious, and generated chimeric virus progeny BJ2A which expressed two fusion proteins, EDIII-2A-CP and EDIII-2A, and one non-recombinant BaMV CP in (**Figures 1C**, left panel, **2**). The chimeric virus BJ2A could infect *N. benthamiana* systemically and produce JEV EDIII throughout whole plants (**Figure 1C**, right panel). The coding sequence of the foreign peptide EDIII-2A was stably maintained in the genome of the chimeric virus BJ2A after nine serial passages in *C. quinoa* leaves (**Figure 2**). The fusion protein EDIII-2A-CP and free-form BaMV CP subunits were able to assemble into filamentous BJ2A CVPs (**Figures 3A–C**). Although these JEV EDIII contain two cysteines, which potentially could cause changes in virion morphology and stability (Li et al., 2007), BJ2A CVPs exhibited the same particle morphology as that of the wild type BaMV’s (**Figures 3A–C**). Furthermore, JEV EDIII antibody could specifically recognize BJ2A CVPs, indicating that the JEV EDIII peptide was properly presented on the surface of BJ2A CVPs (**Figures 3B,C**). Most importantly, BJ2A CVPs elicited immuno-responses in mice to generate neutralizing antibodies against the infection of JEV (**Figure 4** and **Table 1**).

Foot-and-mouth disease virus 2A peptide leads to partial dissociation of the fusion proteins with various efficiency for different fusion constructs (Donnelly et al., 2001). In this study, the incorporation of FMDV 2A peptide allowed production of enough free-form BaMV CP and the EDIII-2A-Cp fusion protein for both the assembly of stable CVPs and proper display of the epitope on the surface. In contrast, the CP produced by the construct pBJ is expected to be the EDIII-CP fusion form only, which might hinder the virion assembly process and result in the loss of infectivity of pBJ (as shown in **Figure 1**). The use of FMDV 2A peptide might have facilitated the virion assembly of the chimeric viruses *in planta* and likely contributed to the maintenance of the foreign coding sequences over long-term successive passages (**Figure 2**). Furthermore, the BaMV virion-based epitope-presentation system might provide an adjuvant-like function (Gerloni et al., 2000; Savard et al., 2011) and compensated for the partial incorporation of the EDIII-2A-CP in the CVPs.

The immunization assays in mice further confirmed that the EDIII peptide was presented in a biologically functional conformation on the surfaces of the CVPs, since the BJ2A CVPs elicited effective immuno-response against JEV infection in mice (**Figure 4** and **Table 1**). These results demonstrated the potential and applicability of the BaMV-based vector system in producing potent vaccine candidates in plants.

### Comparison with Other Plant-Based Vaccine Candidate Producing Systems against JEV or Related Viruses

As mentioned above, plants have been actively explored as effective vaccine candidate-producing systems in recent years. For JEV and related flaviviruses, transgenic and transient expression approaches have been documented (Martinez et al., 2012; Chen and Lai, 2013). JEV subunit vaccine candidate produced in transgenic rice has been reported to elicit antigen-specific neutralizing antibodies in mice previously (Wang et al., 2009). However, the yields of JEV E protein expressed in the leaves of transgenic rice were relatively low, amounting to 1.1–1.9 μg mg^-1^ (corresponding to 0.11–0.19% of TSP) (Wang et al., 2009). For transient expression approach, the EDIII of Dengue virus type 2 (D2EDIII), with only slight differences in structure from that of JEV EDIII (Chavez et al., 2010), was successfully produced in *N.* *benthamiana* using tobacco mosaic virus (TMV)-based duplicated-promoter strategy. The yield of D2EDIII protein accounted for 0.28% of TSP (Saejung et al., 2007). For another closely related virus, West Nile virus (WNV), Chen et al. (2011) developed a virus-like particle (VLP) vaccine by fusing the EDIII of WNV to the C-terminus of hepatitis B core antigen (HBcAg) and utilized a geminivirus-based vector to express the recombinant protein in *N. benthamiana* (Chen et al., 2011). The assembly of the VLP and the effectiveness in inducing strong B and T-cell responses were demonstrated. The yield of the WNV EDIII-HBcAg fusion protein was estimated to be ∼0.35 μg mg^-1^ fresh leaf weight (FLW). By using the MagnICON vector system, the accumulation level was increased to >1 μg mg^-1^ FLW (Chen, 2015). In contrast, the BJ2A virus expressed JEV EDIII-2A-CP fusion proteins in the leaves at levels reaching 8.9 ± 4.3 μg mg^-1^ FLW. Therefore, the BaMV-based vector systems have enabled rapid expression of recombinant proteins at levels comparable to or higher than those produced with previous transgenic or other transient expression approaches in plants.

As for immunogenicity, the plant-made D2EDIII elicited only low level of anti-dengue virus antibodies, and no antibody induction was detected when mice were immunized without adjuvant (Saejung et al., 2007), possibly due to the small size of D2EDIII fragment expressed. In comparison, the BJ2A virus displayed the peptide of interest on the surface of assembled CVPs, enhancing immunogenicity (**Table 1**) by taking advantage of using BaMV CP as the dominant pathogen-derived antigens (Gerloni et al., 2000; Massa et al., 2008). VLPs and CVPs have been known to induce strong protective responses in the absence of adjuvants (Roldao et al., 2010). The repetitive display of the epitopes on the quasi-crystalline surface of CVPs may serve as the prime target for B-cell recognition and trigger strong B-cell responses (Fehr et al., 1998). It has been shown that the immunization by using JEV EDIII can elicit the generation of neutralizing antibodies to protect against JEV infection (Kaur et al., 2002). In this study, we have found that BJ2A CVPs could induce IgG-level immune responses in mice (**Figure 4A**). Moreover, the fluorescence staining results indicated that BJ2A CVPs successfully induced the anti-JEV virus antibody in mice (**Figure 4B**).

As for the preparation of immunogens, the D2EDIII proteins were purified by immobilized metal ion affinity chromatography (Saejung et al., 2007). In this study, the macromolecular nature of BJ2A CVPs allowed for the development of easy procedures for virion purification and the recovery of high doses of recombinant protein by simple centrifugation. Therefore, BaMV-based epitope presentation strategy provides an efficient alternative for convenient, rapid, and low-cost expression of vaccine candidates.

### The Advantages of BaMV-Based Epitope Presentation System

Plants have been explored as bioreactors for the production of therapeutic proteins, and several plant-produced biopharmaceuticals have been through Phases II and III clinical trials in humans (Daniell et al., 2009; Rybicki, 2010, 2014; Thomas et al., 2011; Chen, 2015). It has also been shown that the plant-produced CVPs administered to animals intranasally, intraperitoneally or orally are able to induce strong neutralizing immune responses (Pogue et al., 2002; Rybicki, 2010). In addition, many achievements have been made using plant virus-based vector with FMDV 2A strategy for expressing foreign proteins as vaccines (e.g., Smolenska et al., 1998; O’Brien et al., 2000; Marconi et al., 2006; Zelada et al., 2006; Uhde-Holzem et al., 2010). In this study, we demonstrated that the BaMV-based vector system allowed expression of longer peptide, up to 111 amino acids, on CVPs than *Potato virus X*-based vector did (Marconi et al., 2006; Uhde-Holzem et al., 2010). The BaMV-based vector offered some advantages compared to other available systems. Firstly, BaMV has a narrow host range in nature, and therefore is ecologically safer for field use (Hsu and Lin, 2004), minimizing the concern for environmental contaminations. Secondly, by the incorporation of FMDV 2A peptide, BaMV-based epitope-presentation vector was stable over long-term successive passages, as opposed to the previously described systems (Porta and Lomonossoff, 1998; Porta et al., 2003; Lico et al., 2006). Thirdly, the plant, *C. quinoa*, used for the production of JEV subunit vaccine candidate is a widely cultivated crop (Bhargava et al., 2006), and poses minimal safety concern in animals. Furthermore, we have resolved the atomic model of the BaMV virion structure by using cryo-electron microscopy recently (DiMaio et al., 2015). This model provides the theoretical basis for the modeling of more candidate epitopes to be presented on BaMV-based vector system by using convenient *in silico* analyses.

## Conclusion

To our knowledge, this is the first report describing the production of a vaccine candidate of JEV EDIII using plant virus-based vector system. Our results also demonstrated the feasibility of using FMDV 2A peptide to circumvent some commonly encountered problems for plant virus-based epitope presentation systems. This strategy enabled the production of large quantity of both EDIII-2A-CP fusion protein and free CP in plant cells, allowing the self-assembly of stable CVPs using the two forms of CPs. As compared to the construct pBJ, which does not express non-recombinant BaMV CP, the incorporation of 2A peptide improved the infectivity of the chimeric virus BJ2A and might contribute to the enhanced stability over serial passages and the preservation of key structural features of the CVPs. The BaMV-based CVP vaccine successfully induced the generation of neutralizing antibodies against JEV infection. Together, these results demonstrated that the BaMV-based CVP system may serve as an alternative for the production of effective and useful vaccine candidates against JEV infections.

## Author Contributions

Designed the study: T-HC, C-CH, J-TL, N-SL, Y-LLi, and Y-HH. Analyzed the data: T-HC, J-TL, Y-WH, and Y-LLe. Wrote the manuscript: T-HC, C-CH, and Y-HH. Interpreted the data, and revised the manuscript: C-CH, Y-WH, N-SL, Y-LLi, and Y-HH. All authors read, edited and approved the final manuscript.

## Conflict of Interest Statement

The authors declare that the research was conducted in the absence of any commercial or financial relationships that could be construed as a potential conflict of interest.
